# The impact of self-distancing on emotion explosiveness and accumulation: An fMRI study

**DOI:** 10.1371/journal.pone.0206889

**Published:** 2018-11-06

**Authors:** Maxime Résibois, Jean-Yves Rotgé, Pauline Delaveau, Peter Kuppens, Iven Van Mechelen, Philippe Fossati, Philippe Verduyn

**Affiliations:** 1 Faculty of Psychology and Educational Sciences, KU Leuven, Leuven, Belgium; 2 Inserm U 1127, CNRS UMR 7225, Sorbonne Université, UPMC Univ Paris 06, UMR S 1127, Institut du Cerveau et de la Moelle, ICM-A-IHU, Social and Affective Neuroscience (SAN) Laboratory & Prisme Platform, Paris, France; 3 AP-HP, Department of Psychiatry, Pitié-Salpêtrière Hospital, Paris, France; 4 Centre de NeuroImagerie de Recherche – CENIR, Institut du cerveau et de la moelle (ICM), Sorbonne Universités, UPMC Univ Paris 06, Inserm, CNRS – Hôpital Pitié-Salpêtrière, Paris, France; 5 Faculty of Psychology and Neuroscience, Maastricht University, Maastricht, Netherlands; National Institute of Mental Health, UNITED STATES

## Abstract

Emotions unfold over time with episodes differing in explosiveness (i.e., profiles having a steep vs. a gentle start) and accumulation (i.e., profiles increasing over time vs. going back to baseline). In the present fMRI study, we wanted to replicate and extend previous findings on the psychological and neural mechanisms underlying emotion explosiveness and accumulation. Specifically, we aimed to: (a) replicate the finding that different neural mechanisms are associated with emotion explosiveness and accumulation, (b) replicate the finding that adopting a self-distanced (vs. self-immersed) perspective decreases emotion explosiveness and accumulation at the level of self-report, and (c) examine whether adopting a self-distanced (vs. self-immersed) perspective similarly modulates activity in the brain regions associated with emotion explosiveness and accumulation. Participants in an fMRI scanner were asked to adopt a self-immersed or self-distanced perspective while reading and thinking about negative social feedback, and to report on felt changes in negative affect during that period using an emotion intensity profile tracking approach. We replicated previous findings showing that emotion explosiveness and accumulation were related to activity in regions involved in self-referential processing (such as the medial prefrontal cortex) and sustained visceral arousal (such as the posterior insula), respectively. The finding that adopting a self-distanced (vs. self-immersed) perspective lowers emotion explosiveness and accumulation was also replicated at a self-report level. However, perspective taking did not impact activity in the neural correlates of emotion explosiveness and accumulation.

## Introduction

Emotions are dynamic processes that unfold over time. As such, studying the temporal features of emotions is a prerequisite to reach a full understanding of how emotions function [1,2]. Moreover, the fact that many forms of psychopathology (e.g., depression, post-traumatic stress disorder [3]) are characterized by disturbances in patterns of emotion unfolding only adds to the importance of research on emotion dynamics.

To study the dynamics of single emotion episodes, Frijda and colleagues [4–6] developed an intensity profile tracking approach. This approach consists of asking participants to recollect recent emotional episodes and to draw a curve reflecting continuous changes in emotion intensity during each episode.

In several studies, it has been shown that emotion intensity profiles collected with an intensity profile tracking approach can take a wide range of possible shapes reflecting the inherent complexity of emotion dynamics [1,2,7,8]. To describe this shape variability, Frijda and colleagues used a number of dynamic features, such as the number of peaks and valleys, the intensity of the highest peak, and the area underneath the curve. However, these features were selected in an ad-hoc fashion. To overcome this limitation, Verduyn and colleagues [2] wanted to empirically infer dynamic features that would optimally describe variability in emotion intensity profiles. Using dimension reduction techniques, they found that the two features which explained most variability are emotion explosiveness and accumulation [2,9,10]. Emotion explosiveness reflects whether the profile has a steep versus a gentle start. Emotion accumulation reflects whether the profile increases over time versus goes back to baseline. To better understand variability in profile shapes, one should not only examine which features optimally describe this variability, but also identify the factors influencing these feature [1].

In a recent functional magnetic resonance imaging (fMRI) study, it was found that different neural regions underlie emotion explosiveness and accumulation [11]. In particular, whereas explosiveness was found to be related to regions involved in self-referential processing such as the medial prefrontal cortex (mPFC), accumulation was related to regions underlying sustained visceral arousal such as the posterior insula. These findings are consistent with theoretical claims in the field of emotion dynamics and emotion regulation that emotion onset and offset are partially governed by different processes [12–16]. However, as the study reported in [11] was the first attempt to uncover the neural basis of emotion explosiveness and accumulation, it was largely exploratory in nature and its results need to be replicated, which is also in line with recent calls for more replication studies in the field of fMRI [17].

A further issue that has been investigated is whether the perspective taken by the emotion-experiencing person may impact the emotion’s explosiveness and accumulation. Previous research has indeed found that one way that people deal with emotional events is by reflecting upon them [18], and that two types of self-reflection can be distinguished: adopting a self-immersed (i.e., first-person) or a self-distanced (i.e., third-person or external observer) perspective [19–21]. In contrast to adopting a self-immersed perspective, self-distancing was found to lead individuals to experience decreased levels of emotional and physiological reactivity, intrusive ideation, psychological stress and depressed affects [22–27]. However, previous research on self-distancing largely disregarded the dimension of time, with a notable exception being a study by Verduyn and colleagues [28] who found that adopting a self-distanced perspective shortens the duration of emotional experience. However, these authors did not examine the impact of perspective taking on the shape of emotion intensity unfolding. With regard to this issue, Résibois and colleagues asked participants in a recent study [29] to adopt either a self-immersed or a self-distanced perspective while reflecting upon negative social feedback. Adopting a self-distanced perspective was found to lead to reduced levels of both emotion explosiveness and accumulation as compared to adopting a self-immersed perspective. Unfortunately, however, this study only relied on self-report data and did not examine the possible impact of the perspective manipulation on activity in the neural correlates of the two dynamic features underlying the variability in emotion intensity profiles.

### The present study

The present study is set up to contribute to our understanding of emotion dynamics by replicating and extending previous findings on emotion explosiveness and accumulation with three specific aims. The first aim is to replicate seminal findings on the neural correlates of emotion explosiveness and accumulation. Consistent with Résibois, Verduyn, and colleagues [11], we expect explosiveness to be related to activity in the medial prefrontal cortex and accumulation to activity in the posterior insula. The second aim is to replicate the previously found effect of perspective taking on emotion explosiveness and accumulation at the level of self-report. Consistent with Résibois and colleagues [29], we expect emotional episodes to be characterized by lower levels of both explosiveness and accumulation when participants adopt a self-distanced versus a self-immersed perspective. The third aim is to examine the possible impact of perspective taking on the neural correlates of emotion explosiveness and accumulation. We expect lower activity in the medial prefrontal cortex (associated with explosiveness) and posterior insula (associated with accumulation) when participants adopt a self-distanced as compared to a self-immersed perspective.

To test these hypotheses we make use of an fMRI setup in which we induce negative emotions by means of negative social feedback, and ask participants to adopt a self-immersed or self-distanced perspective while reading and thinking about the feedback. Subsequently, we ask them to report on felt changes in emotion intensity using an intensity profile tracking approach. The perspective instructions, feedback form and intensity profile tracking approach were explained during a short task training. Following the procedure used in Résibois, Verduyn, and colleagues [11], non-negative matrix factorization will be used to decompose the collected intensity profiles into an explosiveness and accumulation component, which, in turn, will be used as regressors of the BOLD signal. Next, we will model the effect of the perspective taking manipulation on emotion explosiveness and accumulation at the level of self-report as well as at the level of the neural correlates of the two dynamic features under study.

## Method

All variables collected in the study are mentioned and we report all experimental conditions.

### Sample

A target number of 40 participants was set prior to the beginning of the study and we slightly oversampled to anticipate participants possibly not showing up at the study. Forty-two French speaking participants (22 females, mean age = 26.45, SD = 7.77, with ages ranging from 18 to 48 years old, all right handed) were thus recruited a month prior to the study through the RISC mailing list of the CNRS (France) that contains more than 10 000 people volunteering to participate in scientific experiments. These 42 participants were screened for any contraindication for MRI such as claustrophobia, metallic prostheses, neurologic or psychiatric illnesses, medication or drugs intake. All participants were found to be eligible and provided written informed consent to participate in the study that took place between May and November 2015. Payment for participation was 45 Euros. A total of ten participants had to be excluded from the analyses due to (a) technical scanner issues (*n* = 2), (b) excessive movement (*n* = 1), (c) disbelief in the cover story (see also funnelled debriefing below, *n* = 6), or (d) being that upset by the feedback that the experiment had to be stopped (*n* = 1). This resulted in a final sample of thirty-two participants (18 females, Mean age = 26.34, SD = 7.67, with ages ranging from 18 to 48 years old). The study was approved by University Paris VI’s institutional review board.

### Materials

#### Social feedback paradigm

Following previous studies [30–32], negative social feedback was used to induce emotions for two reasons: (a) in daily life emotions are often caused by social stimuli [33,34] and (b) social feedback elicits emotional responses that are long enough to study emotion dynamics [35]. The social feedback consisted of ratings on desirable (e.g., interesting, honest) and undesirable (e.g., stubborn, superficial) personality traits as well as on an item assessing whether the evaluator would like to have the participant as a friend (an English translation of the original feedback forms is shown in S1 Supporting Information). Negative feedback consisted of low (high) ratings on desirable (undesirable) items as well as on the evaluator’s desire to have the participant as a friend. Neutral feedback consisted of ratings close to the neutral scale midpoint of all items. Feedback was shown in one of two pre-specified orders, preventing the presentation of more than two consecutive trials of the same valence (negative or neutral), counterbalanced across participants.

#### Perspective taking instructions

Participants were asked to adopt a self-distanced or self-immersed perspective when reading and thinking about the feedback. In the self-distanced perspective condition, participants were instructed to “*read and think about the feedback while adopting a detached attitude with regard to this feedback*, *as if you were an impartial observer*, *a scientist who analyses the feedback objectively*”. In the self-immersed perspective condition, participants were instructed to “*read and think about the feedback while concentrating on what it implies for you as a person*, *on what are the specific feelings you are experiencing subjectively at this feedback*”. These instructions were modelled after previous studies manipulating immersed versus distanced perspective taking [36,37].

#### Emotion intensity profile tracking approach

Immediately after exposure to social feedback, participants drew with a trackball a profile reflecting continuous changes in the intensity of negative affect during the period that they read and thought about the feedback. For this purpose, a two-dimensional grid was displayed on the screen. The *X*-axis represented time and was proportionally divided into two parts corresponding to the period during which participants read (30s) and reflected upon the feedback (60s). The *Y*-axis represented the intensity of negative affect and was divided into seven intervals ranging from ‘none’ to ‘very high’. The intensity labels on the *Y*-axis were identical for self-immersed and self-distanced trials.

#### Task training

To explain participants what the social feedback would look like, ensure that they understood the perspective instructions, and familiarize them with reporting on emotion unfolding using the emotion intensity profile tracking approach, participants were walked through each screen of a practice feedback trial. First, the experimenter clarified the meaning of a self-immersed and a self-distanced perspective and answered any possible questions participants had on these constructs. Next, the items constituting the social feedback were explained using a blank feedback form, and participants were reminded that they had to read the social feedback while adopting the instructed perspective. Then, participants were explained that they had to continue to think about the feedback adopting the instructed perspective as long as a fixation cross appeared on the screen. Finally, the emotion intensity profile tracking approach was explained and participants practiced until they felt capable of drawing emotion intensity profiles.

### Procedure

The experiment was divided in four phases. In phase 1 (20 min), participants wrote four brief texts on personal topics such as “Describe what is most important in your life”. Participants were made to believe that these texts would be read by five evaluators who would use the texts to assess participants’ personality. In reality, no evaluators were involved and all participants received the same feedback. To further strengthen the cover story, participants were told that the supposed evaluators would be misled themselves into thinking that each essay had been written by a different participant, supposedly allowing the experimenter to assess the stability of first impressions.

In phase 2 (20 min), participants completed several questionnaires assessing personality traits, emotion regulation dispositions, and well-being indicators. As these are not directly relevant for our research questions, they will be left aside in the remainder of the manuscript.

**Fig 1:**
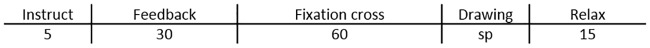
Time course of trials (in seconds). Each trial started with a screen announcing that feedback was about to be shown and reminded participants which perspective (self-immersed or self-distanced) to adopt (Instruct). Subsequently, while adopting the instructed perspective, participants had to read one of the negative (six trials per run) or neutral (four trials per run) feedback that was presented (Feedback), and to think about it while adopting the instructed perspective as long as a fixation cross appeared on the screen (Fixation cross). Immediately afterwards, they were asked to draw an intensity profile reflecting the changes in negative affect they experienced while reading and thinking about the feedback using the emotion intensity profile tracking approach (Drawing). To reduce carryover effects, participants were asked to relax before a new trial started (Relax). sp = self-paced.

In phase 3 (50 min), after a short training, participants entered into the MRI scanner and were exposed to social feedback across two runs consisting of 10 trials each (see Fig 1 for a visual representation of the structure of a trial). At the start of each run, participants were instructed to adopt a self-distanced or self-immersed perspective (manipulated within participants with the order of perspectives counterbalanced across participants). Both conditions thus consisted of the same number of trials (10 each).

Finally, in phase 4 (10 min), participants went through a funnelled debriefing consisting of several questions that offered plenty of opportunities to the participants to express any suspicion they may have had about the veracity of the cover story (for the full list of questions, see S1 Supporting Information). The funnelled debriefing was followed by a full debriefing revealing the true purpose of the experiment.

### Image acquisition

Stimuli were generated and presented with E-Prime 2.0 and projected on a Plexiglas screen mounted at the end of the scanner bore. Two functional runs were acquired on a 3T Siemens MAGNETOM Prisma^fit^ Tim MR-scanner VD 13 (Siemens Medical Solutions, Erlangen, Germany) with Siemens standard 32-channel head coil. Participants’ head movements were restrained by foam paddings inside of the head coil. Functional images covering the whole brain were acquired using a T2*-weighted gradient echo, echo planar imaging (EPI) sequence, sensitive to blood oxygen level-dependent signal, employing the following parameters: repetition time: 2040ms, echo time: 27ms, flip angle: 78°, bandwidth: 2444Hz, matrix: 66×66, field of view: 19.8×19.8cm^2^, GRAPPA acceleration factor: 2. Forty sequential axial slices, with an isotropic voxel size of 3×3×3mm^3^, were acquired parallel to the anteroposterior commissure plane. Each run lasted between 1240s and 1838s (mean = 1395s, SD = 107), resulting in between 608 and 901 images (mean = 684 images, SD = 53) depending on the time participants took to draw emotion intensity profiles. Additional "dummy” volumes were acquired at the beginning of each run to allow the magnetization to stabilize to a steady state before the first real volume. High-resolution three-dimensional T1-weighted sagittal images (3D fast gradient echo inversion recovery sequence, inversion time: 900ms, repetition time: 2300ms, echo time: 2.96ms, bandwidth: 240Hz, flip angle: 9°, matrix: 256×248, field of view: 25.6×25.6cm^2^, voxel size: 1×1×1mm³, GRAPPA acceleration factor: 2) were acquired for anatomical localization.

### Data analysis

Whereas the first author as well as two co-authors were involved in the analysis of the original dataset [11], only one of them analysed the present dataset.

#### Delineating emotion explosiveness and accumulation

Consistent with Résibois, Verduyn, and colleagues [11], each of the obtained 384 self-reported intensity profiles following negative feedback was first transformed into a function using the linear interpolation function (interp1) implemented in MATLAB R2016b [38] and then discretised into 44 equally distanced time points, corresponding to the number of images acquired during the period that participants read and thought about the feedback. These time series were subsequently decomposed into two components using non-negative matrix factorization [39] (as implemented in MATLAB R2016b [38]). The resulting component loadings depict the dynamic features of the shape of component profiles, whereas the resulting component scores depict the extent to which each intensity profile is characterized by each of these dynamic features.

**Fig 2:**
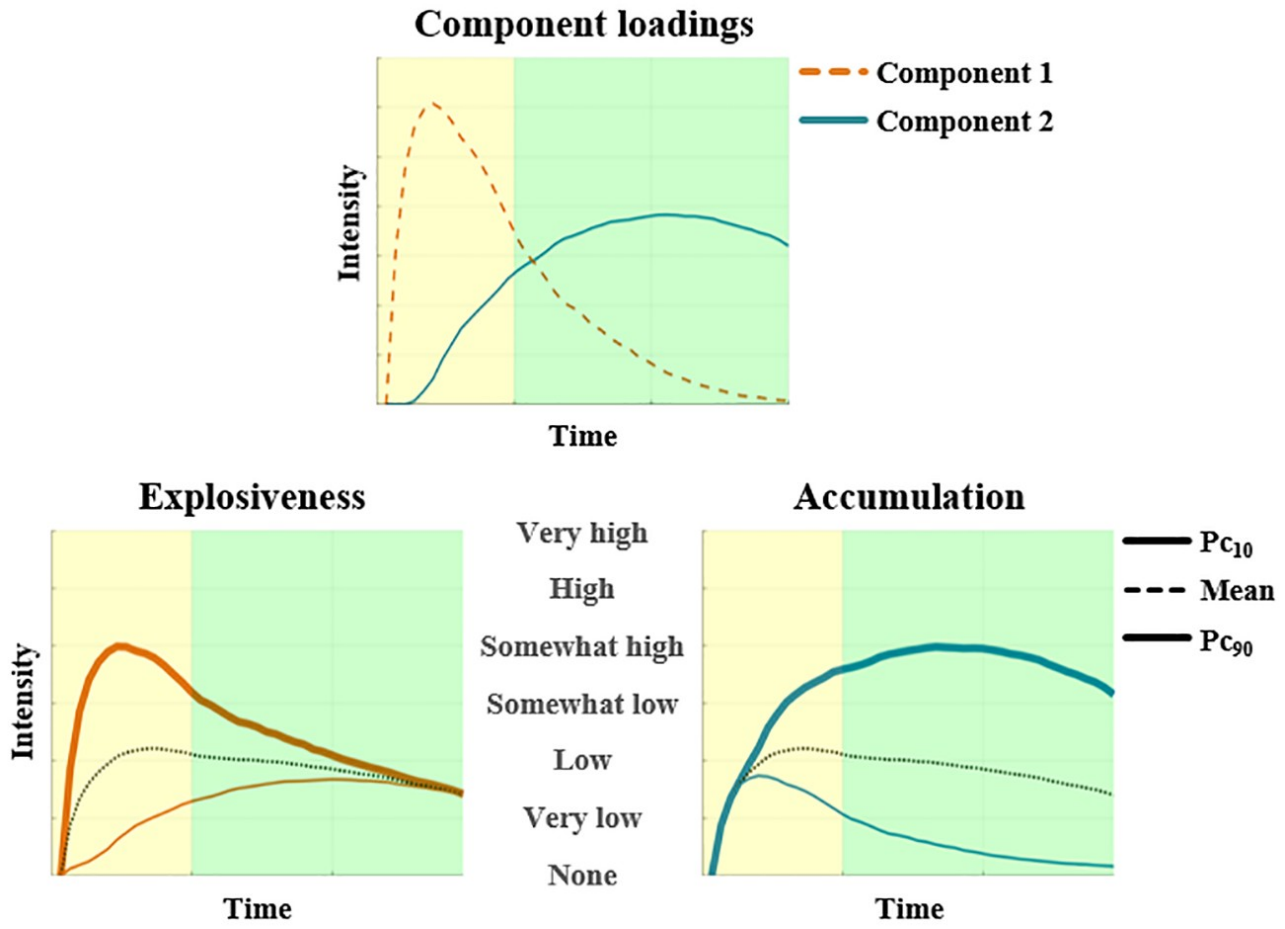
Two-component solution resulting from NNMF. Yellow (left) and green (right) backgrounds correspond to reading and thinking about the feedback, respectively. Top: Component loadings of emotional intensity profiles over time. Bottom: Reconstructed profiles taking a high (90^th^ percentile), average, or low (10^th^ percentile) score on the component in question and a mean score on the other component, presented according to the order of their peaks in the temporal process. This figure is based on data obtained in both self-immersed and self-distanced trials. Bottom left panel: High and low scoring profiles show an explosive and gentle start, respectively. Bottom right panel: High and low scoring profiles show emotion accumulation and recovery, respectively.

As illustrated in Fig 2 (top panel), the first obtained component has initial high loadings followed by a steep decrease, whereas the second obtained component has loadings that increase over time. To interpret these components [40], reconstructed profiles taking low (i.e., 10^th^ percentile), average, and high (i.e., 90^th^ percentile) scores on one component and mean scores on the other component were constructed (see Fig 2, bottom panel). The first component corresponds to emotion explosiveness, with emotion intensity having either a gentle (10^th^ percentile) or an explosive (90^th^ percentile) start. The second component corresponds to emotion accumulation, with emotion intensity either returning to baseline (10^th^ percentile) or accumulating (90^th^ percentile) over time. Reconstructed profiles taking low, average and high scores on one component and mean scores on the other component, separately for each perspective taking condition are available in S1 Fig.

**Fig 3:**
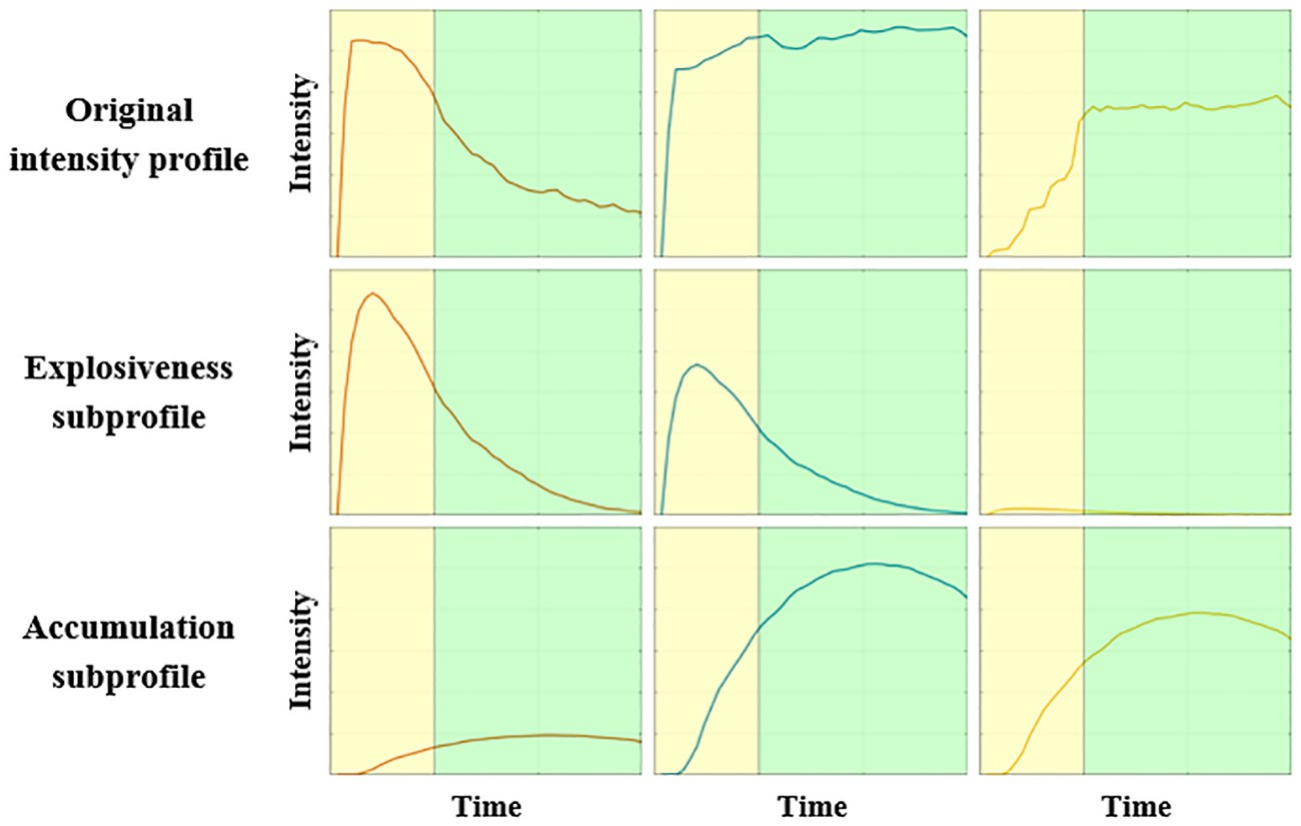
Original drawings (upper panel), explosiveness subprofiles (middle panel) and accumulation subprofiles (lower panel). Adding the reconstructed subprofiles closely approximates the original intensity profile. Yellow (left) and green (right) backgrounds correspond to reading and thinking about the feedback, respectively.

Each intensity profile can thus be reconstructed by summing the component scores multiplied by their corresponding loadings (i.e., adding reconstructed subprofiles). A visualization of the decomposition of intensity profiles into their reconstructed subprofiles is shown in Fig 3.

#### Pre-processing of brain images

Functional scans were pre-processed with SPM8 [41], using slice-time correction, motion correction, spatial normalization to the MNI space, and spatial smoothing using a 8-mm full-width at half-maximum isotropic Gaussian kernel. Spatial normalization was performed by first co-registering the high resolution T1-weighted image to the mean functional image, normalizing the T1 to the MNI template, and applying the normalization parameters to the functional images.

#### General linear model construction

Statistical analyses were conducted using the general linear model (GLM) framework implemented in SPM8 [41]. For each run, boxcar regressors were used to represent the first screen displaying the self-perspective instruction (self-paced). For each trial within each run, boxcar regressors were also used to represent: (a) the five-second screen notifying participants of the forthcoming feedback and reminding them of the perspective to take, (b) the ninety-second period during which participants read and thought about the manipulated negative feedback, (c) the ninety-second period during which participants read and thought about the manipulated neutral feedback, and (d) the self-paced emotion intensity profile drawing period, with the relaxation period functioning as an implicit baseline. All regressors were convolved with the canonical haemodynamic response function. Similar to Résibois, Verduyn, and colleagues [11], a high-pass filter of 200s was applied, and the motion realignment parameters were included as regressors of non-interest.

#### Neural correlates of emotion intensity profile features

To examine the neural basis of emotion explosiveness and accumulation, we further added the reconstructed subprofiles derived from the non-negative matrix factorization (as depicted in Fig 3), convolved with the haemodynamic response function, to the regression equation of the GLM presented above. This model was used to predict the BOLD signal both in a number of regions of interest and at the voxel level, and this across the two perspectives.

Two series of region of interest analysis were conducted to try to replicate earlier results on the neural correlates of emotion explosiveness and accumulation. Specifically, as replicating our previous findings was one of the key motivations for this study, in the first series of region of interest analysis we used two *global* regions of interest that comprised all clusters that were found to be related with emotion explosiveness (resp. emotion accumulation) in the study by Résibois, Verduyn and colleagues [11]. The first region of interest with all clusters found to be related with emotion explosiveness comprised the left mPFC, the left middle and superior frontal and temporal gyri, the left supramarginal gyrus, the right angular, superior temporal, lingual, and middle occipital gyri, and the right cerebellum. The second region of interest with all clusters found to be related with emotion accumulation comprised the bilateral insula (mid-posterior part) and cingulate cortex (mid-posterior part), the right claustrum and anterior cingulate cortex (dorsal part), the left middle frontal (dorsolateral part of the prefrontal cortex), pre/post-central, and superior temporal gyri, the left caudate body, and inferior parietal lobule. These were created by first saving all SPM-8’s clusters from the result table of explosiveness (resp. accumulation) as a binary image, and transforming it into a ROI using the SPM8-compatible tool MarsBar [42]. In the second series, we used the two *specific* regions of interest found by Résibois, Verduyn and colleagues [11] to be correlated with emotion explosiveness and accumulation, respectively (i.e., the mPFC and insula, respectively). The mPFC and insula were bilaterally defined using AAL’s [43] entire structural masks included in the SPM8-compatible tool MarsBar [42], with the insula being divided into an anterior (y > -10) and posterior (y < -10) sub-region [44]. For each region of interest, we calculated the mean value of the second-level explosiveness and accumulation BOLD regression weights by aggregating across all of their voxels, and tested for significance by means of one sample t-tests with Bonferroni correction.

In addition, voxelwise whole brain analyses were conducted to explore possible additional correlates of emotion explosiveness and accumulation. Specifically, we created statistical parametric maps for each participant and entered those into random-effects group analyses testing for significance using one sample *t*-tests. Similar to Résibois, Verduyn, and colleagues [11], statistical parametric maps were thresholded at *p*<.001 (uncorrected) combined with an extend threshold of 10 adjacent voxels, which balances Type I and Type II error rates [45,46]. To test the robustness of our findings, we also provide FWER cluster-corrected and FDR voxelwise-corrected *p*-values. Resulting peaks were transformed into the Talairach space using the SPM8-compatible tool icbm2tal [47,48] and labelled using the Talairach atlas [49,50].

#### The effect of perspective taking on emotion explosiveness and accumulation at the self-report level

To examine the effect of the perspective taking manipulation on emotion explosiveness and accumulation, we ran multilevel analyses using the nlme package [51] developed for R [52]. In particular, the two non-negative matrix factorization scores (i.e., explosiveness and accumulation) were predicted by a dummy predictor (0 = self-immersion, 1 = self-distancing) at Level 1. The intercept and slope were allowed to vary randomly across participants.

#### The effect of perspective taking on the neural correlates of emotion explosiveness and accumulation

To examine whether perspective taking impacts activity in the neural correlates of emotion explosiveness and accumulation, two contrasts were created based on the parameters obtained from fitting the general linear model described above without the reconstructed subprofiles (i.e., only containing boxcar regressors). The contrasts compared neural activity during the period that participants adopted a self-distanced (SD) perspective to the period that participants adopted a self-immersed (SI) perspective and vice versa. Both contrasts were examined at the level of region of interests as well as voxels across the whole brain.

## Results

### Neural correlates of emotion intensity profile features

The two reconstructed subprofiles (i.e., component loadings multiplied by component scores, see Fig 3) of explosiveness and accumulation (convolved with the canonical haemodynamic response function) were used as regressors of the BOLD response across the two conditions. First, we examined whether we could replicate the findings of Résibois, Verduyn and colleagues [11] by conducting region of interest analyses. Next, we explored possible additional neural correlates by conducting voxelwise whole brain analyses. It is notable that the neural correlates of explosiveness and accumulation did not depend on the type of self-perspective adopted. Indeed, contrasts comparing the neural correlates of explosiveness (accumulation) while adopting a self-distanced perspective to the neural correlates of explosiveness (accumulation) while adopting a self-immersed perspective were not significant, regardless of whether conducting voxelwise whole-brain analysis or region of interest analyses.

In a first series of region of interest analyses we examined whether the current explosiveness and accumulation regressors were predictive of neural activity in the *global* regions of interest that comprised all clusters identified by Résibois, Verduyn and colleagues [11] to be associated with emotion explosiveness and accumulation, respectively. This was found to be the case (see Table 1).

**Table 1:** Region of interest analyses predicting neural activity in the full set of clusters observed by Résibois, Verduyn & colleagues [11] to be correlated with emotion explosiveness (resp. accumulation).

Region of interest	Explosiveness		Accumulation	
	T	*p*	T	*p*
Full set of clusters^a^ that appeared to be correlated in [11] with explosiveness	3.54	.001	-.18	.82
Full set of clusters^b^ that appeared to be correlated in [11] with accumulation	-4.95	1.00	3.56	.001

*p*-values are Bonferroni-corrected for multiple testing;

^a^left mPFC, left middle and superior frontal and temporal gyri, left supramarginal gyrus, right angular, superior temporal, lingual, and middle occipital gyri, and right cerebellum;

^b^bilateral insula (mid-posterior part) and cingulate cortex (mid-posterior part), right claustrum and anterior cingulate cortex (dorsal part), left middle frontal (dorsolateral part of the prefrontal cortex), pre/post-central, and superior temporal gyri, left caudate body, and inferior parietal lobule.

In a second series of region of interest analyses we examined whether the explosiveness and accumulation regressors were predictive of neural activity in the *specific* regions identified by Resibois, Verduyn and colleagues [11] to be associated with emotion explosiveness (mPFC) and accumulation (posterior insula), respectively. This was found to be the case (see Table 2).

**Table 2:** Region of interest analyses predicting neural activity in the *specific* regions observed by Resibois, Verduyn & colleagues [11] to underlie emotion explosiveness (resp. accumulation).

ROI	Explosiveness		Accumulation	
	T	*p*	T	*p*
mPFC	2.49	.03	1.90	.10
Insula				
*Anterior*	-.71	.99	.73	.55
*Posterior*	-3.19	1.00	3.18	.005

mPFC = medial prefrontal cortex. *p*-values are Bonferroni-corrected for multiple testing.

**Fig 4:**
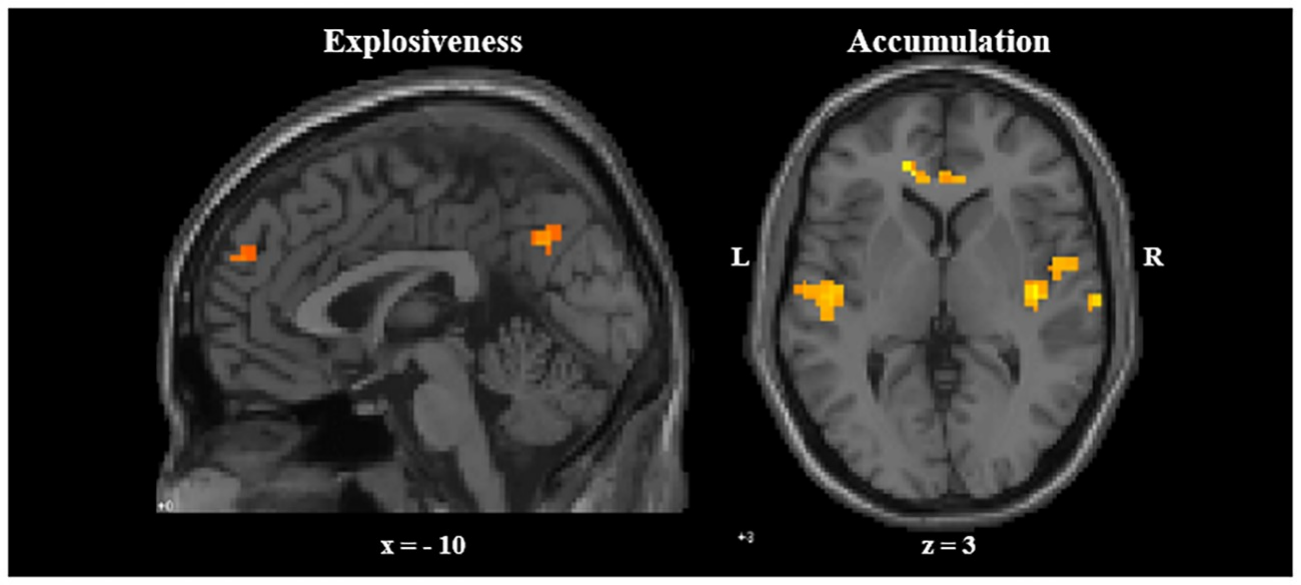
Neural correlates of emotion explosiveness and accumulation. Left panel: mPFC activation associated with emotion explosiveness. Right panel: Insula activation associated with emotion accumulation. Coordinates in the Talairach space.

Finally, in line with previous findings [11], voxelwise whole brain analyses revealed that emotion explosiveness is related to activity in the medial prefrontal cortex, the bilateral middle frontal and superior temporal gyri, the left middle temporal gyrus, and the right middle occipital gyrus. However, a number of regions were additionally identified with explosiveness also being related to activity in left inferior and right frontal gyri, the right middle temporal gyrus, the left precentral, middle occipital, and lingual gyri, the bilateral cuneus, and the right precuneus (See Table 3 and Fig 4).

**Table 3:** Activations associated with explosiveness in whole-brain analysis.

			Tal coordinates (mm)	
Region of activation	BA	T value	[x;y;z]	Vox.
				20
L. Medial Frontal Gyrus	6	3.65†	[-1;48;34]	
				193*
L. Inferior Frontal Gyrus	9	6.14†	[-57;12;24]	
L. Middle Frontal Gyrus	6	4.61†	[-40;4;48]	
L. Precentral Gyrus	6	3.81†	[-35;-2;31]	
				154*
R. Middle Frontal Gyrus	46	5.04†	[54;23;25]	
R. Middle Frontal Gyrus	9	4.30†	[40;20;32]	
R. Middle Frontal Gyrus	6	4.29†	[34;4;52]	
				47
R. Middle Frontal Gyrus	47	4.36†	[41;38;-7]	
R. Inferior Frontal Gyrus	10	4.01†	[44;45;2]	
				34
R. Superior Frontal Gyrus	8	4.04†	[7;35;46]	
				36
L. Inferior Frontal Gyrus	45	3.99†	[-51;34;5]	
				533*
R. Middle Occipital Gyrus	18	6.65†	[26;-93;5]	
R. Cuneus	17	6.20†	[10;-96;4]	
R. Cuneus	18	6.18†	[18;-96;7]	
				951*
L. Cuneus	18	6.46†	[-24;-95;-2]	
L. Middle Occipital Gyrus	18	5.68†	[-29;-91;10]	
L. Lingual Gyrus	18	5.42†	[-18;-86;-9]	
				967*
L. Superior Temporal Gyrus	39	5.63†	[-46;-58;26]	
L. Middle Temporal Gyrus	39	5.12†	[-27;-64;28]	
R. Precuneus	7	4.99†	[23;-59;32]	
				32
R. Superior Temporal Gyrus	38	4.74†	[44;14;-28]	
R. Middle Temporal Gyrus	21	4.27†	[55;5;-20]	
				26
L. Superior Temporal Gyrus	38	3.92†	[-45;20;-23]	
L. Middle Temporal Gyrus	21	3.77†	[-51;9;-25]	
L. Middle Temporal Gyrus	21	3.71†	[-56;5;-17]	

All *p*s<.001 uncorrected, number of voxels>10 per cluster.

BA = Brodmann’s areas. Vox. = Voxels per cluster. L = left. R = right.

^†^p<.05 voxelwise FDR-corrected.

*p<.05 FWE-corrected at cluster level.

Likewise, in line with previous findings [11], exploratory voxelwise whole brain analyses revealed that emotion accumulation is related to activity in the bilateral posterior insula, the left precentral, cingulate, middle frontal and superior temporal gyri, the left caudate body, and the right claustrum. However, a number of regions were additionally identified with accumulation also being related to activity in the left middle temporal, anterior cingulate, superior and medial frontal gyri, the left precuneus, the right paracentral lobule and caudate tail, the right middle and superior temporal gyri, and the bilateral angular gyri (See Table 4 and Fig 4).

**Table 4:** Activations associated with accumulation in whole-brain analysis.

Region of activation	BA	T value	Tal coordinates (mm)	Vox.
			[x;y;z]	
				18
R. Insula	13	4.01†	[32;-10;21]	
R. Insula	13	3.97†	[29;-22;25]	
				835*
L. Precentral Gyrus	6	5.77†	[-43;-7;20]	
R. Paracentral Lobule	31	5.10†	[4;-29;46]	
L. Precentral Gyrus	4	4.72†	[-27;-27;48]	
				42
L. Anterior Cingulate	32	4.54†	[-12;33;11]	
L. Anterior Cingulate	24	4.29†	[-1;31;5]	
				14
L. Caudate Body		4.15†	[-9;16;20]	
L. Cingulate Gyrus	24	3.56†	[-4;18;26]	
				16
L. Caudate Body		3.99†	[-15;-28;29]	
L. Cingulate Gyrus	31	3.96†	[-18;-36;26]	
				12
R. Caudate Tail		4.75†	[21;-43;12]	
				49
L. Superior Frontal Gyrus	8	4.56†	[-15;24;45]	
L. Medial Frontal Gyrus	8	4.06†	[-10;30;46]	
				27
L. Middle Frontal Gyrus	6	4.50†	[-35;13;46]	
				23
L. Superior Frontal Gyrus	9	4.42†	[-15;46;28]	
				19
L. Angular Gyrus	39	4.33†	[-54;-59;34]	
				30
R. Angular Gyrus	39	4.13†	[54;-59;33]	
R. Middle Temporal Gyrus	39	4.09†	[51;-67;27]	
R. Superior Temporal Gyrus	39	3.88†	[57;-58;20]	
				324*
R. Superior Temporal Gyrus	42	5.49†	[63;-29;14]	
R. Superior Temporal Gyrus	41	4.56†	[38;-32;16]	
R. Claustrum		4.38†	[38;-17;4]	
				12
L. Superior Temporal Gyrus	22	4.20†	[-57;-60;15]	
L. Middle Temporal Gyrus	39	3.72†	[-54;-66;20]	
				38
L. Precuneus	19	4.63†	[-41;-76;35]	

All *p*s<.001 uncorrected, number of voxels>10 per cluster.

BA = Brodmann’s areas. Vox. = Voxels per cluster. L = left. R = right.

^†^p<.05 voxelwise FDR-corrected.

*p<.05 FWE-corrected at cluster level.

### The effect of perspective taking on emotion explosiveness and accumulation at the self-report level

Multilevel analysis was used to predict emotion explosiveness and accumulation scores by the perspective taking manipulation (0 = self-immersed, 1 = self-distanced). Both explosiveness (B = -174.88, β = -.34, t(351) = -3.79, p < .001, 95% confidence interval [CI] [-265.70, -84.05]) and accumulation (B = -301.46, β = -.33, t(351) = -3.35, p < .001, 95% CI [-478.65, -124.26]) were found to be lower when participants adopted a self-distanced perspective. The manipulation order was not related to either explosiveness (p = .28) or accumulation (p = .97), nor did controlling for the manipulation order alter any of the reported conclusions.

### The effect of perspective taking on the neural correlates of emotion explosiveness and accumulation

Adopting a self-distanced (vs. self-immersed) perspective did not lead (see Table 5) to altered levels of activity in the mPFC (associated with explosiveness) or in the insula (associated with accumulation). Additional exploratory voxelwise whole-brain analyses similarly did not reveal differential neural activity depending on the self-perspective adopted. An alternative strategy to examine the neural activity associated with adopting a self-distanced (SD) versus a self-immersed (SI) perspective would be to use the neutral trials of the corresponding run as reference categories as reflected by the following two contrasts: (1) [negative trials > neutral trials]_SD_ > [negative trials > neutral trials]_SI_ and (2) [negative trials > neutral trials]_SD_ < [negative trials > neutral trials]_SI_. Yet, similar to the analyses reported above, this did not lead to any significant result in region of interest or in whole brain analyses.

**Table 5:** Regions of interest analyses comparing activity while adopting a self-distanced vs. self-immersed perspective when exposed to negative feedback.

Label	SD > SI		SI > SD	
	T	*p*	T	*p*
mPFC	1.61	.17	-1.61	1.00
Insula				
*Anterior*	.51	.67	-.51	.97
*Posterior*	.45	.70	-.45	.96

SD = self-distancing. SI = self-immersion. mPFC = medial prefrontal cortex.

*p*-values are Bonferroni-corrected for multiple testing.

## Discussion

The overall aim of the present study was to replicate and extend previous results on the psychological and neural mechanisms underlying emotion explosiveness and accumulation. First of all and importantly, our findings provided an independent replication of the existence of distinctive correlates of emotion explosiveness and emotion accumulation with data being acquired by another experimenter and using another fMRI scanner than in the study of Résibois, Verduyn, and colleagues [11]. Specifically, in the two series of region of interest analyses and in the voxelwise whole brain analyses, emotion explosiveness appeared again to be associated with regions of self-referential processing (such as the medial prefrontal cortex), whereas emotion accumulation appeared again to be associated with regions of sustained monitoring of visceral arousal and the sensory component of social exclusion (such as the posterior insula). These findings corroborate that onset- and offset-bound processes have distinct neural correlates, which is consistent with emotion dynamic frameworks distinguishing between two key emotion unfolding phases [10–14,16]: an onset phase (associated with explosiveness) and an offset phase (associated with accumulation), which were found in the present study to be the two main constituents underlying change in emotional experience over time. It further emphasizes the need to take temporal dynamics into account when studying the neural basis of emotions, which resonates with recent calls to put time on the research agenda of affective neuroscience [53–55].

Providing further evidence for an association between emotion explosiveness and self-referential processing, in the current study explosiveness was additionally found to be associated with activity in the precuneus and the temporo-parietal junction, which are two regions of the default-mode network [56–58]. Moreover, as further evidence for an association between emotion accumulation and sustained monitoring of visceral arousal, emotion accumulation additionally appeared to be associated with activity in the primary sensory cortex [54,59,60]. In addition, emotion accumulation was also associated with the dorsal anterior cingulate cortex, providing suggestive evidence for the social feedback having resulted in feelings of social exclusion, even though it should be noted that no significant activity in the anterior insula was observed [61]. However, future studies using non-social or more basic emotion-eliciting stimuli (e.g., emotional pictures as used in [62]) are needed to examine the degree to which the neural correlates of explosiveness and accumulation generalize across contexts. It is possible that studies using such more basic stimuli would identify primary emotion areas such as the amygdala or the anterior insula as key neural correlates of emotion explosiveness and accumulation [63–65]. This is especially likely given the negative association found in the literature between meaning making regions (associated with explosiveness in the present study), and the anterior insula and amygdala [62]. Yet, one could alternatively argue that activity in the anterior insula and amygdala is perhaps associated with emotion intensity per se regardless of the stage of emotion unfolding (i.e., overall levels of intensity rather than specific dynamic features) as found in previous research using emotional movies to induce emotion [66,67]. However, it is notable that, in the present study where we used complex social stimuli, we did not find an effect of our manipulation on any primary emotion areas despite that self-distancing was found to overall lower self-reported emotion intensity.

Second, we indeed replicated that adopting a self-distanced (vs. a self-immersed) perspective decreases both emotion explosiveness and accumulation (i.e., lowering overall emotion intensity). This demonstrates that perspective taking can modulate how people initially react to emotional stimuli, at least when being instructed to use this regulation strategy beforehand. Moreover, it suggests support to psychological therapies that provide people the tools to take distance, including cognitive-behavioural, acceptance-based, and mindfulness therapies [68], such that they may be better equipped to deal with potential future stressors. However, future research including clinical populations and using stimuli eliciting higher degrees of distress are necessary to justify this conjecture.

Third, unexpectedly, adopting a self-distanced (vs. a self-immersed) perspective did not lead to altered levels of activity in the mPFC (associated with explosiveness) or in the insula (associated with accumulation). Although it is difficult to interpret null findings, one could at least speculate that this result might be due to the fact that perspective taking may recruit regions that are also correlated with emotion explosiveness and accumulation, cancelling out the possibility of finding a significant decrease of activity in these regions when adopting a self-distanced (vs. a self-immersed) perspective. This tentative explanation could be especially plausible to at least partially explain why adopting a self-distanced perspective was not found to be associated with a decreased activity in the medial prefrontal cortex (associated with explosiveness), as this region has been consistently found to underlie processes of reappraisal [69–72], including perspective taking [73,74]. This interpretation might especially hold given the use of social feedback as an emotion-eliciting stimulus. The predictive and reactive control systems (PARCS) framework [75] indeed theorizes that regions of the predictive system, including the DMN, underlie both (a) adopting an observer perspective and (b) making meaning of negative feedback that challenges internal models about oneself and the world. Thus, future studies might benefit from using emotional stimuli that do not require extensive self-referential processing (e.g., emotional pictures) to further explore the role of perspective taking on the neural correlates of explosiveness and accumulation.

Finally, although intensity levels reported in the present study are similar to other studies using a negative feedback procedure [10,11,31,32], future research eliciting stronger emotions are needed to get a better understanding of the neural mechanisms underlying emotion dynamics.

## Supporting information

S1 Supporting Information (DOCX)

S1 Fig Two-component solution resulting from NNMF, separately for each perspective taking condition. (TIF)

S1 Dataset Emotion intensity profiles as drawn by participants in E-Prime. (CSV)

S2 Dataset Reconstructed explosiveness subprofiles used as BOLD regressors. (CSV)

S3 Dataset Reconstructed accumulation subprofiles used as BOLD regressors. (CSV)

S1 File Binary mask of regions of interests used in ROI analyses. (ZIP)
