# Supplementary material for: The impact of self-distancing on emotion explosiveness and accumulation: An fMRI study
Source: PLoS One. 2018 Nov 6;13(11):e0206889. doi: 10.1371/journal.pone.0206889 (PMC6219793; doi:10.1371/journal.pone.0206889)
Supplement: S1 Supporting Information — (DOCX) [file pone.0206889.s001.docx]

# S1 Supporting Information

## Negative social feedback forms

| Estimate the author’s personality: | | | | | | | | | | | | | |  |
| --- | --- | --- | --- | --- | --- | --- | --- | --- | --- | --- | --- | --- | --- | --- |
|  | Not at all | | |  | | Neutral | | | | Totally | | | |  |
| Social | | 1 | 2 | | 3 | | 4 | 5 | 6 | | 7 | |  | |
| Stubborn | | 1 | 2 | | 3 | | 4 | 5 | 6 | | 7 | |  | |
| Superficial | | 1 | 2 | | 3 | | 4 | 5 | 6 | | 7 | |  | |
| Interesting | | 1 | 2 | | 3 | | 4 | 5 | 6 | | 7 | |  | |
| Naïve | | 1 | 2 | | 3 | | 4 | 5 | 6 | | 7 | |  | |
| Honest | | 1 | 2 | | 3 | | 4 | 5 | 6 | | 7 | |  | |
|  | | | | | | | | | | | |  | |  |
| Would you like to have as a friend someone like the author? | | | | | | | | | | | | | |  |
|  | Not at all | | |  | | Neutral | | | | Totally | | | |  |
|  | | 1 | 2 | | 3 | | 4 | 5 | 6 | | 7 | |  | |

| Estimate the author’s personality: | | | | | | | | | | | | | |  |
| --- | --- | --- | --- | --- | --- | --- | --- | --- | --- | --- | --- | --- | --- | --- |
|  | Not at all | | |  | | Neutral | | | | Totally | | | |  |
| Social | | 1 | 2 | | 3 | | 4 | 5 | 6 | | 7 | |  | |
| Stubborn | | 1 | 2 | | 3 | | 4 | 5 | 6 | | 7 | |  | |
| Superficial | | 1 | 2 | | 3 | | 4 | 5 | 6 | | 7 | |  | |
| Interesting | | 1 | 2 | | 3 | | 4 | 5 | 6 | | 7 | |  | |
| Naïve | | 1 | 2 | | 3 | | 4 | 5 | 6 | | 7 | |  | |
| Honest | | 1 | 2 | | 3 | | 4 | 5 | 6 | | 7 | |  | |
|  | | | | | | | | | | | |  | |  |
| Would you like to have as a friend someone like the author? | | | | | | | | | | | | | |  |
|  | Not at all | | |  | | Neutral | | | | Totally | | | |  |
|  | | 1 | 2 | | 3 | | 4 | 5 | 6 | | 7 | |  | |

| Estimate the author’s personality: | | | | | | | | | | | | | |  |
| --- | --- | --- | --- | --- | --- | --- | --- | --- | --- | --- | --- | --- | --- | --- |
|  | Not at all | | |  | | Neutral | | | | Totally | | | |  |
| Social | | 1 | 2 | | 3 | | 4 | 5 | 6 | | 7 | |  | |
| Stubborn | | 1 | 2 | | 3 | | 4 | 5 | 6 | | 7 | |  | |
| Superficial | | 1 | 2 | | 3 | | 4 | 5 | 6 | | 7 | |  | |
| Interesting | | 1 | 2 | | 3 | | 4 | 5 | 6 | | 7 | |  | |
| Naïve | | 1 | 2 | | 3 | | 4 | 5 | 6 | | 7 | |  | |
| Honest | | 1 | 2 | | 3 | | 4 | 5 | 6 | | 7 | |  | |
|  | | | | | | | | | | | |  | |  |
| Would you like to have as a friend someone like the author? | | | | | | | | | | | | | |  |
|  | Not at all | | |  | | Neutral | | | | Totally | | | |  |
|  | | 1 | 2 | | 3 | | 4 | 5 | 6 | | 7 | |  | |

| Estimate the author’s personality: | | | | | | | | | | | | | |  |
| --- | --- | --- | --- | --- | --- | --- | --- | --- | --- | --- | --- | --- | --- | --- |
|  | Not at all | | |  | | Neutral | | | | Totally | | | |  |
| Social | | 1 | 2 | | 3 | | 4 | 5 | 6 | | 7 | |  | |
| Stubborn | | 1 | 2 | | 3 | | 4 | 5 | 6 | | 7 | |  | |
| Superficial | | 1 | 2 | | 3 | | 4 | 5 | 6 | | 7 | |  | |
| Interesting | | 1 | 2 | | 3 | | 4 | 5 | 6 | | 7 | |  | |
| Naïve | | 1 | 2 | | 3 | | 4 | 5 | 6 | | 7 | |  | |
| Honest | | 1 | 2 | | 3 | | 4 | 5 | 6 | | 7 | |  | |
|  | | | | | | | | | | | |  | |  |
| Would you like to have as a friend someone like the author? | | | | | | | | | | | | | |  |
|  | Not at all | | |  | | Neutral | | | | Totally | | | |  |
|  | | 1 | 2 | | 3 | | 4 | 5 | 6 | | 7 | |  | |

| Estimate the author’s personality: | | | | | | | | | | | | | |  |
| --- | --- | --- | --- | --- | --- | --- | --- | --- | --- | --- | --- | --- | --- | --- |
|  | Not at all | | |  | | Neutral | | | | Totally | | | |  |
| Social | | 1 | 2 | | 3 | | 4 | 5 | 6 | | 7 | |  | |
| Stubborn | | 1 | 2 | | 3 | | 4 | 5 | 6 | | 7 | |  | |
| Superficial | | 1 | 2 | | 3 | | 4 | 5 | 6 | | 7 | |  | |
| Interesting | | 1 | 2 | | 3 | | 4 | 5 | 6 | | 7 | |  | |
| Naïve | | 1 | 2 | | 3 | | 4 | 5 | 6 | | 7 | |  | |
| Honest | | 1 | 2 | | 3 | | 4 | 5 | 6 | | 7 | |  | |
|  | | | | | | | | | | | |  | |  |
| Would you like to have as a friend someone like the author? | | | | | | | | | | | | | |  |
|  | Not at all | | |  | | Neutral | | | | Totally | | | |  |
|  | | 1 | 2 | | 3 | | 4 | 5 | 6 | | 7 | |  | |

| Estimate the author’s personality: | | | | | | | | | | | | | |  |
| --- | --- | --- | --- | --- | --- | --- | --- | --- | --- | --- | --- | --- | --- | --- |
|  | Not at all | | |  | | Neutral | | | | Totally | | | |  |
| Social | | 1 | 2 | | 3 | | 4 | 5 | 6 | | 7 | |  | |
| Stubborn | | 1 | 2 | | 3 | | 4 | 5 | 6 | | 7 | |  | |
| Superficial | | 1 | 2 | | 3 | | 4 | 5 | 6 | | 7 | |  | |
| Interesting | | 1 | 2 | | 3 | | 4 | 5 | 6 | | 7 | |  | |
| Naïve | | 1 | 2 | | 3 | | 4 | 5 | 6 | | 7 | |  | |
| Honest | | 1 | 2 | | 3 | | 4 | 5 | 6 | | 7 | |  | |
|  | | | | | | | | | | | |  | |  |
| Would you like to have as a friend someone like the author? | | | | | | | | | | | | | |  |
|  | Not at all | | |  | | Neutral | | | | Totally | | | |  |
|  | | 1 | 2 | | 3 | | 4 | 5 | 6 | | 7 | |  | |

| Estimate the author’s personality: | | | | | | | | | | | | | |  |
| --- | --- | --- | --- | --- | --- | --- | --- | --- | --- | --- | --- | --- | --- | --- |
|  | Not at all | | |  | | Neutral | | | | Totally | | | |  |
| Social | | 1 | 2 | | 3 | | 4 | 5 | 6 | | 7 | |  | |
| Stubborn | | 1 | 2 | | 3 | | 4 | 5 | 6 | | 7 | |  | |
| Superficial | | 1 | 2 | | 3 | | 4 | 5 | 6 | | 7 | |  | |
| Interesting | | 1 | 2 | | 3 | | 4 | 5 | 6 | | 7 | |  | |
| Naïve | | 1 | 2 | | 3 | | 4 | 5 | 6 | | 7 | |  | |
| Honest | | 1 | 2 | | 3 | | 4 | 5 | 6 | | 7 | |  | |
|  | | | | | | | | | | | |  | |  |
| Would you like to have as a friend someone like the author? | | | | | | | | | | | | | |  |
|  | Not at all | | |  | | Neutral | | | | Totally | | | |  |
|  | | 1 | 2 | | 3 | | 4 | 5 | 6 | | 7 | |  | |

| Estimate the author’s personality: | | | | | | | | | | | | | |  |
| --- | --- | --- | --- | --- | --- | --- | --- | --- | --- | --- | --- | --- | --- | --- |
|  | Not at all | | |  | | Neutral | | | | Totally | | | |  |
| Social | | 1 | 2 | | 3 | | 4 | 5 | 6 | | 7 | |  | |
| Stubborn | | 1 | 2 | | 3 | | 4 | 5 | 6 | | 7 | |  | |
| Superficial | | 1 | 2 | | 3 | | 4 | 5 | 6 | | 7 | |  | |
| Interesting | | 1 | 2 | | 3 | | 4 | 5 | 6 | | 7 | |  | |
| Naïve | | 1 | 2 | | 3 | | 4 | 5 | 6 | | 7 | |  | |
| Honest | | 1 | 2 | | 3 | | 4 | 5 | 6 | | 7 | |  | |
|  | | | | | | | | | | | |  | |  |
| Would you like to have as a friend someone like the author? | | | | | | | | | | | | | |  |
|  | Not at all | | |  | | Neutral | | | | Totally | | | |  |
|  | | 1 | 2 | | 3 | | 4 | 5 | 6 | | 7 | |  | |

| Estimate the author’s personality: | | | | | | | | | | | | | |  |
| --- | --- | --- | --- | --- | --- | --- | --- | --- | --- | --- | --- | --- | --- | --- |
|  | Not at all | | |  | | Neutral | | | | Totally | | | |  |
| Social | | 1 | 2 | | 3 | | 4 | 5 | 6 | | 7 | |  | |
| Stubborn | | 1 | 2 | | 3 | | 4 | 5 | 6 | | 7 | |  | |
| Superficial | | 1 | 2 | | 3 | | 4 | 5 | 6 | | 7 | |  | |
| Interesting | | 1 | 2 | | 3 | | 4 | 5 | 6 | | 7 | |  | |
| Naïve | | 1 | 2 | | 3 | | 4 | 5 | 6 | | 7 | |  | |
| Honest | | 1 | 2 | | 3 | | 4 | 5 | 6 | | 7 | |  | |
|  | | | | | | | | | | | |  | |  |
| Would you like to have as a friend someone like the author? | | | | | | | | | | | | | |  |
|  | Not at all | | |  | | Neutral | | | | Totally | | | |  |
|  | | 1 | 2 | | 3 | | 4 | 5 | 6 | | 7 | |  | |

| Estimate the author’s personality: | | | | | | | | | | | | | |  |
| --- | --- | --- | --- | --- | --- | --- | --- | --- | --- | --- | --- | --- | --- | --- |
|  | Not at all | | |  | | Neutral | | | | Totally | | | |  |
| Social | | 1 | 2 | | 3 | | 4 | 5 | 6 | | 7 | |  | |
| Stubborn | | 1 | 2 | | 3 | | 4 | 5 | 6 | | 7 | |  | |
| Superficial | | 1 | 2 | | 3 | | 4 | 5 | 6 | | 7 | |  | |
| Interesting | | 1 | 2 | | 3 | | 4 | 5 | 6 | | 7 | |  | |
| Naïve | | 1 | 2 | | 3 | | 4 | 5 | 6 | | 7 | |  | |
| Honest | | 1 | 2 | | 3 | | 4 | 5 | 6 | | 7 | |  | |
|  | | | | | | | | | | | |  | |  |
| Would you like to have as a friend someone like the author? | | | | | | | | | | | | | |  |
|  | Not at all | | |  | | Neutral | | | | Totally | | | |  |
|  | | 1 | 2 | | 3 | | 4 | 5 | 6 | | 7 | |  | |

| Estimate the author’s personality: | | | | | | | | | | | | | |  |
| --- | --- | --- | --- | --- | --- | --- | --- | --- | --- | --- | --- | --- | --- | --- |
|  | Not at all | | |  | | Neutral | | | | Totally | | | |  |
| Social | | 1 | 2 | | 3 | | 4 | 5 | 6 | | 7 | |  | |
| Stubborn | | 1 | 2 | | 3 | | 4 | 5 | 6 | | 7 | |  | |
| Superficial | | 1 | 2 | | 3 | | 4 | 5 | 6 | | 7 | |  | |
| Interesting | | 1 | 2 | | 3 | | 4 | 5 | 6 | | 7 | |  | |
| Naïve | | 1 | 2 | | 3 | | 4 | 5 | 6 | | 7 | |  | |
| Honest | | 1 | 2 | | 3 | | 4 | 5 | 6 | | 7 | |  | |
|  | | | | | | | | | | | |  | |  |
| Would you like to have as a friend someone like the author? | | | | | | | | | | | | | |  |
|  | Not at all | | |  | | Neutral | | | | Totally | | | |  |
|  | | 1 | 2 | | 3 | | 4 | 5 | 6 | | 7 | |  | |

| Estimate the author’s personality: | | | | | | | | | | | | | |  |
| --- | --- | --- | --- | --- | --- | --- | --- | --- | --- | --- | --- | --- | --- | --- |
|  | Not at all | | |  | | Neutral | | | | Totally | | | |  |
| Social | | 1 | 2 | | 3 | | 4 | 5 | 6 | | 7 | |  | |
| Stubborn | | 1 | 2 | | 3 | | 4 | 5 | 6 | | 7 | |  | |
| Superficial | | 1 | 2 | | 3 | | 4 | 5 | 6 | | 7 | |  | |
| Interesting | | 1 | 2 | | 3 | | 4 | 5 | 6 | | 7 | |  | |
| Naïve | | 1 | 2 | | 3 | | 4 | 5 | 6 | | 7 | |  | |
| Honest | | 1 | 2 | | 3 | | 4 | 5 | 6 | | 7 | |  | |
|  | | | | | | | | | | | |  | |  |
| Would you like to have as a friend someone like the author? | | | | | | | | | | | | | |  |
|  | Not at all | | |  | | Neutral | | | | Totally | | | |  |
|  | | 1 | 2 | | 3 | | 4 | 5 | 6 | | 7 | |  | |

## Neutral social feedback forms feedback

| Estimate the author’s personality: | | | | | | | | | | | | | |  |
| --- | --- | --- | --- | --- | --- | --- | --- | --- | --- | --- | --- | --- | --- | --- |
|  | Not at all | | |  | | Neutral | | | | Totally | | | |  |
| Social | | 1 | 2 | | 3 | | 4 | 5 | 6 | | 7 | |  | |
| Stubborn | | 1 | 2 | | 3 | | 4 | 5 | 6 | | 7 | |  | |
| Superficial | | 1 | 2 | | 3 | | 4 | 5 | 6 | | 7 | |  | |
| Interesting | | 1 | 2 | | 3 | | 4 | 5 | 6 | | 7 | |  | |
| Naïve | | 1 | 2 | | 3 | | 4 | 5 | 6 | | 7 | |  | |
| Honest | | 1 | 2 | | 3 | | 4 | 5 | 6 | | 7 | |  | |
|  | | | | | | | | | | | |  | |  |
| Would you like to have as a friend someone like the author? | | | | | | | | | | | | | |  |
|  | Not at all | | |  | | Neutral | | | | Totally | | | |  |
|  | | 1 | 2 | | 3 | | 4 | 5 | 6 | | 7 | |  | |

| Estimate the author’s personality: | | | | | | | | | | | | | |  |
| --- | --- | --- | --- | --- | --- | --- | --- | --- | --- | --- | --- | --- | --- | --- |
|  | Not at all | | |  | | Neutral | | | | Totally | | | |  |
| Social | | 1 | 2 | | 3 | | 4 | 5 | 6 | | 7 | |  | |
| Stubborn | | 1 | 2 | | 3 | | 4 | 5 | 6 | | 7 | |  | |
| Superficial | | 1 | 2 | | 3 | | 4 | 5 | 6 | | 7 | |  | |
| Interesting | | 1 | 2 | | 3 | | 4 | 5 | 6 | | 7 | |  | |
| Naïve | | 1 | 2 | | 3 | | 4 | 5 | 6 | | 7 | |  | |
| Honest | | 1 | 2 | | 3 | | 4 | 5 | 6 | | 7 | |  | |
|  | | | | | | | | | | | |  | |  |
| Would you like to have as a friend someone like the author? | | | | | | | | | | | | | |  |
|  | Not at all | | |  | | Neutral | | | | Totally | | | |  |
|  | | 1 | 2 | | 3 | | 4 | 5 | 6 | | 7 | |  | |

| Estimate the author’s personality: | | | | | | | | | | | | | |  |
| --- | --- | --- | --- | --- | --- | --- | --- | --- | --- | --- | --- | --- | --- | --- |
|  | Not at all | | |  | | Neutral | | | | Totally | | | |  |
| Social | | 1 | 2 | | 3 | | 4 | 5 | 6 | | 7 | |  | |
| Stubborn | | 1 | 2 | | 3 | | 4 | 5 | 6 | | 7 | |  | |
| Superficial | | 1 | 2 | | 3 | | 4 | 5 | 6 | | 7 | |  | |
| Interesting | | 1 | 2 | | 3 | | 4 | 5 | 6 | | 7 | |  | |
| Naïve | | 1 | 2 | | 3 | | 4 | 5 | 6 | | 7 | |  | |
| Honest | | 1 | 2 | | 3 | | 4 | 5 | 6 | | 7 | |  | |
|  | | | | | | | | | | | |  | |  |
| Would you like to have as a friend someone like the author? | | | | | | | | | | | | | |  |
|  | Not at all | | |  | | Neutral | | | | Totally | | | |  |
|  | | 1 | 2 | | 3 | | 4 | 5 | 6 | | 7 | |  | |

| Estimate the author’s personality: | | | | | | | | | | | | | |  |
| --- | --- | --- | --- | --- | --- | --- | --- | --- | --- | --- | --- | --- | --- | --- |
|  | Not at all | | |  | | Neutral | | | | Totally | | | |  |
| Social | | 1 | 2 | | 3 | | 4 | 5 | 6 | | 7 | |  | |
| Stubborn | | 1 | 2 | | 3 | | 4 | 5 | 6 | | 7 | |  | |
| Superficial | | 1 | 2 | | 3 | | 4 | 5 | 6 | | 7 | |  | |
| Interesting | | 1 | 2 | | 3 | | 4 | 5 | 6 | | 7 | |  | |
| Naïve | | 1 | 2 | | 3 | | 4 | 5 | 6 | | 7 | |  | |
| Honest | | 1 | 2 | | 3 | | 4 | 5 | 6 | | 7 | |  | |
|  | | | | | | | | | | | |  | |  |
| Would you like to have as a friend someone like the author? | | | | | | | | | | | | | |  |
|  | Not at all | | |  | | Neutral | | | | Totally | | | |  |
|  | | 1 | 2 | | 3 | | 4 | 5 | 6 | | 7 | |  | |

| Estimate the author’s personality: | | | | | | | | | | | | | |  |
| --- | --- | --- | --- | --- | --- | --- | --- | --- | --- | --- | --- | --- | --- | --- |
|  | Not at all | | |  | | Neutral | | | | Totally | | | |  |
| Social | | 1 | 2 | | 3 | | 4 | 5 | 6 | | 7 | |  | |
| Stubborn | | 1 | 2 | | 3 | | 4 | 5 | 6 | | 7 | |  | |
| Superficial | | 1 | 2 | | 3 | | 4 | 5 | 6 | | 7 | |  | |
| Interesting | | 1 | 2 | | 3 | | 4 | 5 | 6 | | 7 | |  | |
| Naïve | | 1 | 2 | | 3 | | 4 | 5 | 6 | | 7 | |  | |
| Honest | | 1 | 2 | | 3 | | 4 | 5 | 6 | | 7 | |  | |
|  | | | | | | | | | | | |  | |  |
| Would you like to have as a friend someone like the author? | | | | | | | | | | | | | |  |
|  | Not at all | | |  | | Neutral | | | | Totally | | | |  |
|  | | 1 | 2 | | 3 | | 4 | 5 | 6 | | 7 | |  | |

| Estimate the author’s personality: | | | | | | | | | | | | | |  |
| --- | --- | --- | --- | --- | --- | --- | --- | --- | --- | --- | --- | --- | --- | --- |
|  | Not at all | | |  | | Neutral | | | | Totally | | | |  |
| Social | | 1 | 2 | | 3 | | 4 | 5 | 6 | | 7 | |  | |
| Stubborn | | 1 | 2 | | 3 | | 4 | 5 | 6 | | 7 | |  | |
| Superficial | | 1 | 2 | | 3 | | 4 | 5 | 6 | | 7 | |  | |
| Interesting | | 1 | 2 | | 3 | | 4 | 5 | 6 | | 7 | |  | |
| Naïve | | 1 | 2 | | 3 | | 4 | 5 | 6 | | 7 | |  | |
| Honest | | 1 | 2 | | 3 | | 4 | 5 | 6 | | 7 | |  | |
|  | | | | | | | | | | | |  | |  |
| Would you like to have as a friend someone like the author? | | | | | | | | | | | | | |  |
|  | Not at all | | |  | | Neutral | | | | Totally | | | |  |
|  | | 1 | 2 | | 3 | | 4 | 5 | 6 | | 7 | |  | |

| Estimate the author’s personality: | | | | | | | | | | | | | |  |
| --- | --- | --- | --- | --- | --- | --- | --- | --- | --- | --- | --- | --- | --- | --- |
|  | Not at all | | |  | | Neutral | | | | Totally | | | |  |
| Social | | 1 | 2 | | 3 | | 4 | 5 | 6 | | 7 | |  | |
| Stubborn | | 1 | 2 | | 3 | | 4 | 5 | 6 | | 7 | |  | |
| Superficial | | 1 | 2 | | 3 | | 4 | 5 | 6 | | 7 | |  | |
| Interesting | | 1 | 2 | | 3 | | 4 | 5 | 6 | | 7 | |  | |
| Naïve | | 1 | 2 | | 3 | | 4 | 5 | 6 | | 7 | |  | |
| Honest | | 1 | 2 | | 3 | | 4 | 5 | 6 | | 7 | |  | |
|  | | | | | | | | | | | |  | |  |
| Would you like to have as a friend someone like the author? | | | | | | | | | | | | | |  |
|  | Not at all | | |  | | Neutral | | | | Totally | | | |  |
|  | | 1 | 2 | | 3 | | 4 | 5 | 6 | | 7 | |  | |

| Estimate the author’s personality: | | | | | | | | | | | | | |  |
| --- | --- | --- | --- | --- | --- | --- | --- | --- | --- | --- | --- | --- | --- | --- |
|  | Not at all | | |  | | Neutral | | | | Totally | | | |  |
| Social | | 1 | 2 | | 3 | | 4 | 5 | 6 | | 7 | |  | |
| Stubborn | | 1 | 2 | | 3 | | 4 | 5 | 6 | | 7 | |  | |
| Superficial | | 1 | 2 | | 3 | | 4 | 5 | 6 | | 7 | |  | |
| Interesting | | 1 | 2 | | 3 | | 4 | 5 | 6 | | 7 | |  | |
| Naïve | | 1 | 2 | | 3 | | 4 | 5 | 6 | | 7 | |  | |
| Honest | | 1 | 2 | | 3 | | 4 | 5 | 6 | | 7 | |  | |
|  | | | | | | | | | | | |  | |  |
| Would you like to have as a friend someone like the author? | | | | | | | | | | | | | |  |
|  | Not at all | | |  | | Neutral | | | | Totally | | | |  |
|  | | 1 | 2 | | 3 | | 4 | 5 | 6 | | 7 | |  | |

## Funnelled debriefing questions

The funnelled debriefing questions, always asked in the same order, were as follows:

1. Was everything clear?

2. Was the task easy or difficult?

3. Which aspects were easy of difficult?

(If not already spontaneously mentioned we further asked: Was it easy or difficult to adopt each of the perspective?)

4. Did the two perspectives to be adopted led to differences? If so, which ones?

(If not already spontaneously mentioned we further asked: Did they lead to differences in your emotional experience?)

5. Did you notice important differences between the pieces of feedback? If so, which ones?

6. What was your general impression regarding these pieces of feedback?

(If not already spontaneously mentioned we further asked: Were they fair? Were they rather positive or negative? What kind of emotion did they elicit?)

7. Would you like to meet the judges? Why (not)?
