# Supplementary figures and images for: The impact of self-distancing on emotion explosiveness and accumulation: An fMRI study
Source: PLoS One. 2018 Nov 6;13(11):e0206889. doi: 10.1371/journal.pone.0206889 (PMC6219793; doi:10.1371/journal.pone.0206889)

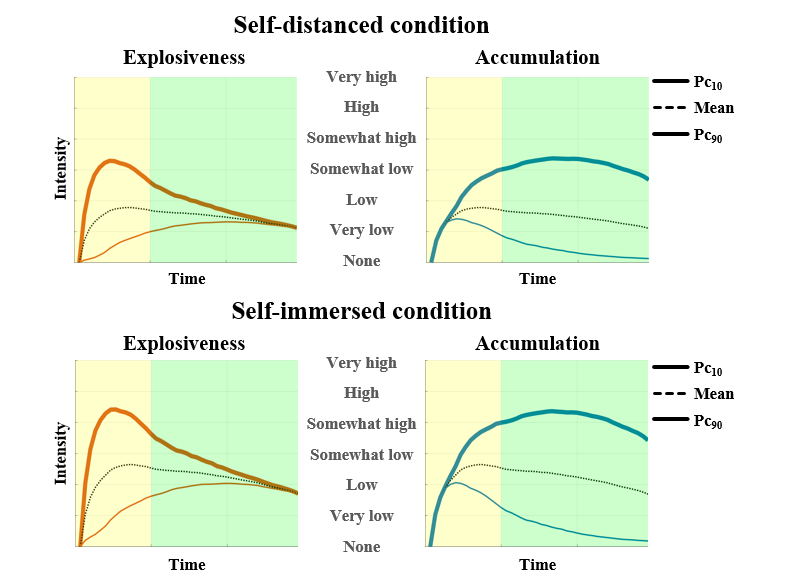

Supplement: S1 Fig — (TIF) [file pone.0206889.s002.tif]
